# A Novel Combination of *Galega officinalis* Extract, *Hippophae rhamnoides* Fruit Oil and Resveratrol Attenuates Bleomycin-Induced Senescence Markers in Human Dermal Fibroblasts and Ex Vivo Hair Follicles

**DOI:** 10.3390/cimb48101041

**Published:** 2026-10-08

**Authors:** Ana Lobo Aldezabal, Irene Cáceres Estévez, Marta Escobar Gómez Rodulfo, Sandra Fernández-Martos, María Calvo-Sánchez, Luisa Haya Rodríguez, Ana López Sánchez, Luis Nieto Botella, Manuel Asín, José Manuel Carbajo, Jesús Espada Regalado

**Affiliations:** 1 Instituto de Investigaciones Biosanitarias, Universidad Francisco de Vitoria, 28223 Madrid, Spain; ana.lobo@ufv.es (A.L.A.); marta.escobar@ufv.es (M.E.G.R.); sandra.fernandezmar@ufv.es (S.F.-M.); maria.calvo@ufv.es (M.C.-S.); 2 Cantabria Labs, 28043 Madrid, Spain; irene.caceres@cantabrialabs.es (I.C.E.); luisa.haya@cantabrialabs.es (L.H.R.); ana.lopezsanchez@cantabrialabs.es (A.L.S.); luis.nieto@cantabrialabs.es (L.N.B.); 3 Skintarget BD (Biological Developments), Parque Científico de Madrid, Universidad Autónoma de Madrid, 28049 Madrid, Spain; 4 Independent Researcher, 03001 Alicante, Spain; galeno.47@hotmail.com; 5 Medical Hydrology Group, Department of Radiology, Rehabilitation and Physiology, Complutense University of Madrid, 28040 Madrid, Spain; jocarbaj@ucm.es

**Keywords:** *Galega officinalis*, *Hippophae rhamnoides*, resveratrol, senescence, resilience, hair follicle

## Abstract

Senescence is an adaptive state activated in cells when external or internal damage exceeds the homeostatic capacity of background resilience mechanisms. It is characterized by a stable cell-cycle arrest, chromatin reorganization, and the acquisition of a senescence-associated secretory phenotype (SASP). While senescence can have beneficial roles in tissue repair, the accumulation of senescent cells contributes to inflammation, stem cell dysfunction, and tissue degeneration. In this context, the identification of compounds able to modulate the threshold between tissue resilience and cellular senescence, by partially eliminating senescent cells to preserve tissue function, is a major target of biomedical research. Here, we present a novel ingredient consisting of a combination of *Galega officinalis* extract, *Hippophae rhamnoides* fruit oil, and resveratrol (Metforhair^®^, MFH). We report that MFH partially reduces senescence-associated features, using human primary dermal fibroblasts as an experimental validation model. Consequently, we used ex vivo cultures of human hair follicles to evaluate the ability of MFH to attenuate senescence markers. Our results support the use of MFH as a hair follicle therapy, which modestly reduces hallmarks of cellular senescence.

## 1. Introduction

Cellular senescence is a stable state of cell cycle arrest that occurs in response to diverse stressors, including telomere shortening, DNA damage, oxidative stress, oncogene activation, and exposure to cytotoxic agents [1]. Although senescent cells no longer proliferate, they remain metabolically active and undergo profound molecular and phenotypic changes [2,3]. These include activation of tumor suppressor pathways such as the p53–p21 and p16INK4a–retinoblastoma (RB) axes, persistent DNA damage signaling, chromatin remodeling, and increased lysosomal activity, frequently detected through senescence-associated *β*-galactosidase (SA-*β*-gal) activity [4,5]. A hallmark feature of senescent cells is the development of the senescence-associated secretory phenotype (SASP), characterized by the secretion of pro-inflammatory cytokines, growth factors, and proteases that can influence neighboring cells and tissue microenvironments [4].

The production of senescent cells occurs regardless of the age of the tissue. The accumulation of these cells is more commonly associated with aging tissues, which severely affects the cellular renewal balance, limiting the resilience potential of the tissue [6,7]. The clearance of these senescent cells enhances the resilient response of the tissue to cope with sustained damage [8]. In this sense, the search for compounds capable of attenuating cellular senescence has become a social priority, and particularly relevant in dermocosmetics.

The current limited availability of molecules, particularly natural compounds of botanical origin capable of effectively targeting the senescent phenotype, has boosted the search for new active ingredients showing efficacy [9]. Here we present a novel combination of *Galega officinalis* extract, *Hippophae rhamnoides* fruit oil, and resveratrol formulated under the registered name Metforhair^®^ (MFH). It seems to partially eliminate senescent cells in a human primary dermal fibroblast model. As well as in ex vivo grown human hair follicles (HFs), both models were previously induced to enter a DNA-damage senescent state. The induction of senescence was performed by Bleomycin (Bleo), an acute genotoxic model.

*Galega officinalis* is a leguminous perennial herb [10], rich in guanidine [11], from which the known antidiabetic molecule metformin is derived [12]. Notably, it has been reported that metformin can induce autophagy and hair regeneration in male and female mice [13]. *Hippophae rhamnoides*, or sea buckthorn, is a flowering shrub [14], showing anti-inflammatory, antioxidant, antimicrobial, and neuroprotective effects [15]. It is also recognized for its rejuvenating, anti-scarring, and anti-wrinkle properties in skin [14]. Resveratrol is a polyphenol, showing outstanding antioxidant, anti-tumor, and anti-aging properties in different experimental models [16]. Our approach combines these three components, considering that this strategy has generally proven effective in dermocosmetic applications [17].

Here we report the characterization of a novel combination of natural ingredients (MFH) as a promising senescent-attenuating agent. First, human primary dermal fibroblasts were used as an experimental validation model. Then, ex vivo cultures of human hair follicles were used as mini-organs to evaluate the effect of MFH on senescence markers. The study of the effect of MFH at a cellular level, and its translation to an ex vivo model, allows us to consider its impact on the hair of patients. Our results suggest that MFH could contribute to the clearance of accumulated senescent cells.

## 2. Materials and Methods

### 2.1. Experimental Working Formulation of Metforhair^®^

The cosmetic ingredient supplied by Cantabria Labs (Madrid, Spain) under the commercial name Metforhair^®^ (INCI: *Galega Officinalis* Extract, *Resveratrol*, *Hippophae Rhamnoides* Fruit Oil) was prepared to ensure suitability for in vitro assays. All compounds used complied with the supplier’s quality specifications and were considered suitable for the intended experimental use. A hydroalcoholic stock solution of the ingredient was prepared containing 0.66% (*v*/*v*) ethanol (absolute ethanol, 131085.1214, PanReac AppliChem, Barcelona, Spain) and adjusted to a final concentration of 10 mg mL^−1^. Prior to experimental use, the stock solution was filtered through a 0.45 µM pore PTFE membrane filter (Millipore, Sigma, Burlington, MO, USA). The specific quantitative ratios of the individual components are subject to a patent protection process and therefore not disclosed here. For treatments of cultured primary fibroblasts and hair follicles, the stock solution was diluted 1/100 (0.1 mg/mL) and 1/10 (1 mg/mL), respectively, in the corresponding culture media (Section 2.2).

### 2.2. Cell Culture

Human dermal fibroblasts were obtained from human skin remnants of cosmetic surgery interventions following previously described protocols [18]. The donors were under 40 years old. Cells were first subjected to mechanical dissociation followed by enzymatic digestion with collagenase A. Cells were maintained in culture using DMEM supplemented with 10% FBS, 1× L-glutamine, and 1× antibiotic–antimycotic solution (all from Gibco Life Technologies, Carlsbad, CA, USA) at 37 °C in a humidified atmosphere with 5% CO_2_.

#### 2.2.1. Cell Senescence Induction

For senescence evaluation in cell cultures using bleomycin (Bleo) (Sigma-Aldrich, St. Louis, MO, USA), 40,000 cells were seeded into Nunc multiwell 6 plates (Sigma-Aldrich). Cells were exposed to Bleo (10 µM) or mock treatment for 24 h. After this period, the medium was replaced, and MFH was added to the culture medium at a final concentration of 0.1 mg/mL (Section 2.1) when indicated. Then cells were maintained for 6 days under standard culture conditions to allow senescence to develop (Figure S1A). The experimental conditions included: control, Bleo (10 µM) for 24 h, Mock treatment for 24 h followed by MFH (0.1 mg/mL), and Bleo (10 µM) for 24 h followed by MFH (0.1 mg/mL).

#### 2.2.2. Cell Biomass Quantification by Crystal Violet Staining

Quantification of adherent cells (primary human fibroblast cultures) was assessed using crystal violet staining. For crystal violet staining, cells were fixed with cold methanol at the end of the experiment [19,20]. After fixation, cells were briefly washed with PBS and incubated for 40 min with 0.1% crystal violet staining solution. The staining solution was then removed, and cells were carefully washed with running water and allowed to dry overnight. Once dried, the dye was solubilized in 10% acetic acid, and absorbance was measured at 580 nm. Cell viability was calculated relative to the control cells seeded in the same multiwell plate as the treated cells.

#### 2.2.3. SA-β-Galactosidase Assay

Cellular senescence was assessed by senescence-associated β-galactosidase (SA-*β*-gal) staining following the classical protocol described [21]. Cells were fixed with 2% formaldehyde and 0.2% glutaraldehyde in PBS and then incubated with staining solution containing 1 mg/mL X-gal substrate (20 mg/mL stock), 40 mM citric acid/sodium phosphate buffer (pH 6.0), 5 mM potassium ferrocyanide, 5 mM potassium ferricyanide, 150 mM NaCl, and 2 mM MgCl_2_ overnight at 37 °C in the dark. Cells were mounted using Fluoromount™ with DAPI (both from Sigma-Aldrich) and visualized using a Nikon Eclipse Ci microscope (Nikon Corporation, Tokyo, Japan) coupled to a Jenoptik PROGRES GRYPHAX^®^ SUBRA Super HD camera (Jenoptik PROGRES GRYPHAX^®^ SUBRA Super HD camera).

#### 2.2.4. Characterization of Senescence Cell Clearance and Proliferation Assays

For immunological detection of target proteins in human dermal fibroblasts, cell cultures (Section 2.2.1) were fixed in cold methanol, and cells were permeabilized with 0.5% Triton X-100. Non-specific binding sites were blocked using BSA. Samples were then incubated overnight at 4 °C in a humidified chamber with the primary antibodies against anti-Ki-67 (ab16667, Abcam, Cambridge, UK) and anti-p21 (Mouse Anti-p21 antibody, clone SX118, 556430, BD Pharmingen^TM^, BD Biosciences, San Jose, CA, USA). After washing with PBS, cells were incubated with the appropriate secondary antibody (Goat anti-Rabbit IgG (H + L) Highly Cross-Adsorbed Secondary Antibody, Alexa Fluor™ 546—A-11035; Goat anti-Mouse IgG (H + L) Cross-Adsorbed Secondary Antibody, Alexa Fluor™ 488—A-11001; Invitrogen, Carlsbad, CA, USA) and DAPI (100 ng/mL). Finally, samples were mounted using Pro-Long mounting medium (Invitrogen) and analyzed using a THUNDER microscope (Leica DMi8; Leica, Wetzlar, Germany).

The TUNEL detection kit (Roche Diagnostics GmbH, Mannheim, Germany) was used according to the instructions of the manufacturer, with the objective of observing partial cell elimination. Image acquisition was performed using a Nikon Eclipse Ci microscope (Nikon Corporation, Tokyo, Japan) coupled to a Jenoptik PROGRES GRYPHAX^®^ SUBRA Super HD camera (Jenoptik PROGRES GRYPHAX^®^ SUBRA Super HD camera).

### 2.3. Hair Follicle Culture

Human hair follicles (HFs) were obtained from human scalp samples taken from the occipital region of volunteer donors, under 40 years old, during a hair transplant procedure. HFs in each experimental series come from different patients. For each patient, several follicles are used and divided equally amongst the different conditions under consideration. This occurs in each series. Therefore, the sample size (*n*) for each trial accounts for the variability within the population comprising different individuals. Eligible patients provided written informed consent. The selected HFs were mature anagen follicles (approximately anagen VI–VII). HFs were not selected on the basis of an assumed hair-cycle stage. Prior to their inclusion in the experiments, individual follicles were examined by phase-contrast microscopy and selected according to a morphostructural assessment aimed at verifying their overall integrity and preservation of the main follicular structures. Follicles showing evident structural damage or morphological deterioration were excluded from the experiments.

HFs were cultured in Williams E medium (Sigma-Aldrich) supplemented with 10× Penicillin/Streptomycin, 10× Amphotericin B, and 2 mM L-glutamine, 5 µg/mL Insulin, 5 µg/mL transferrin, 20 pM T3 hormone, 0.083 µg/mL cholera toxin, and 0.4 µg/mL hydrocortisone (all from Gibco) at 37 °C in a 5% CO_2_ humidified atmosphere. Adherent tissue remnants in HFs were maintained throughout the ex vivo growth process to improve hair follicle viability in basal conditions. HFs from the same individual and body location were used in each experimental series.

#### 2.3.1. Hair Follicle Senescence Induction

For senescence induction in HFs, experimental conditions included: control, Bleo (10 µM), and Bleo (10 µM) + MFH (1 mg/mL). HFs were treated with Bleo (10 µM) or mock treatment for 24 h; after this period, the medium was replaced to remove the treatment. Afterwards, MFH was added to the Bleo + MFH group at a final concentration of 1 mg/mL (from Section 2.1). All HFs (without changing the medium) were subsequently maintained in culture for up to 6 additional days to allow the development of senescence and the SASP [22] (Figure S1B). Bright-field images were acquired at selected time points to monitor morphological changes.

#### 2.3.2. Histological Analysis

For histological analysis, HFs were fixed in 3.7% aqueous pH-neutral formaldehyde and embedded in paraffin using standard procedures (after following Section 2.3.1). Typically, 18–22, 6 µm thick, longitudinal tissue sections were obtained for each HF. As a rule, the 4–6 most central sections, encompassing as much as possible the full length of at least, the suprabulbar fiber and hair bulb regions, were used for comparative analysis. Remnant tissue sections were used to implement immunological procedures. To analyse morphological changes at tissue level, histologically equivalent sections were evaluated by standard H&E staining. Bright-field image acquisition was performed using a Nikon Eclipse Ci (Nikon, Tokyo, Japan) coupled to a Jenoptik PROGRES GRYPHAX^®^ SUBRA Super HD camera and suited Version 1.1.8.153 image software pack (Jenoptik AG, Jena, Germany).

#### 2.3.3. Monitoring Cellular Changes by LDH Release in Hair Follicles

To evaluate the cytotoxicity of the MFH treatment as well as Bleo, an LDH assay was performed using the commercial CyQUANT™ LDH Cytotoxicity Assay Kit (Invitrogen, Thermo Fisher Scientific, Waltham, MA, USA). Supernatants were collected from HF samples after 6 days of MFH treatment following Bleo exposure (Section 2.3.1). LDH activity released into the culture medium was measured by analyzing each supernatant from each experiment individually, following the manufacturer’s instructions. For LDH activity quantification, absorbance values measured at 680 nm were subtracted from those obtained at 490 nm, and cytotoxicity was calculated using the formula: % cytotoxicity = ((Experimental condition LDH activity − control LDH activity)/(maximum LDH activity − control LDH activity)) × 100. Maximum LDH activity was determined by incubating one HF with the lysis buffer provided in the kit for 45 min and was analyzed in parallel with the remaining samples. Within each experiment, values from treated samples were normalized to the corresponding control samples from the same donor to reduce inter-individual variability.

#### 2.3.4. RNA Extraction and Gene Expression Analysis

For gene expression analysis, total RNA from 15 to 20 HFs (from Section 2.3.1) used in each experimental condition (Control, Bleo, Bleo + MFH) was extracted using the RNeasy micro kit (Qiagen, Hilden, Germany) following the instructions of the manufacturer. RNA from each experimental condition was converted into cDNA using the FastGene Scriptase II cDNA Kit (Nippon Genetics, Tokyo, Japan). cDNA quality, quantification, normalization, and subsequent gene expression analysis by qRT-PCR were performed by the Genomic Service of the Madrid Scientific Park (Parque Científico de Madrid) using a QuantStudio 12K Flex system (ThermoFisher, Waltham, MA, USA) and SYBR Green primers. qRT-PCR data were analyzed using a comparative CT method, using *GAPDH* RNA expression as an internal control. Relative gene expression changes were represented as 2^−ΔCt^ values, normalized to the reference gene *GAPDH* and compared between treated and control HFs. Analyzed genes included *p53*, *CDKN1A* (p21), *CDKN2A* (p16), *IL-6*, *ANKRD1*, *CCND1* and *LMNB1*. The sequences used are listed below in Table 1.

A total of three independent (*n* = 3) experiments were analyzed by qRT-PCR. In each independent experiment, the same number of HFs (15 to 20) were used in each experimental condition sample, and amplifications were performed in duplicate for each sample. Gene expression values in each duplicate experimental condition sample were normalized to the control condition to reduce inter-individual variability.

### 2.4. Cell Proliferation and Structural Integrity of HFs

An ELISA assay was performed to evaluate the cell proliferation marker Ki-67 in HFs (from Section 2.3.1). The commercial Human Ki-67 Protein (Ki-67) ELISA Kit (Biorbyt, Cambridge, UK) was used, following the instructions of the manufacturer. Four independent experiments were analyzed, using a minimum of 10 HFs per experimental condition and always maintaining the same number of follicles across experimental conditions. The standard curve provided with the kit was used as a positive control, with known Ki-67 concentrations of 20, 10, 5, 2.5, 1.25, 0.63, 0.32, and 0 ng/mL. Ki-67 levels in the samples were calculated by extrapolating the absorbance values measured at 450 nm using the standard curve generated with the kit standards. Within each experiment, values from treated samples were normalized to the corresponding control samples from the same donor to reduce inter-individual variability.

For immunological detection of target structural proteins in HFs, histological tissue sections were used (Section 2.3.1). HFs were fixed in 3.7% formaldehyde and embedded in paraffin as described. Antigen retrieval was performed using 10 mM citrate buffer in hydrated sections following standard procedure. Primary antibodies, anti-E-Cadherin (610182, Clone 36/E-Cadherin, Waters, MA, USA), were incubated overnight at 4 °C in a wet chamber, washed with PBS, incubated for 1 h at room temperature with appropriate secondary fluorescence antibodies and DAPI (100 ng/mL), and mounted in ProLong and analyzed using a THUNDER microscope (Leica DMi8).

### 2.5. Senescence Cell Clearance in Hair Follicles

TUNEL detection kit (Roche) was used according to the instructions of the manufacturer for apoptotic HFs (from Section 2.3.1) staining. Image acquisition was performed using a Nikon Eclipse Ci (Nikon, Tokyo, Japan) coupled to a Jenoptik PROGRES GRYPHAX^®^ SUBRA Super HD camera and suited Version 1.1.8.153 image software pack (Jenoptik AG, Jena, Germany).

Annexin V was also selected as a target to evaluate the induction of apoptosis in HF (from Section 2.3.1) protein extracts. For this purpose, the Human Annexin V SimpleStep ELISA^®^ Kit (ab223863, Abcam, Cambridge, UK) was used according to the manufacturer’s instructions. Four independent experiments were analyzed, using a minimum of 15 HFs in each experimental condition and always maintaining the same number of follicles across conditions. The standard curve provided in the kit was used as a positive control, with the following Annexin V concentrations: 3000, 1500, 750, 375, 187.5, 93.75, 46.88, and 0 pg/mL. Annexin V concentrations in the samples were determined by extrapolating the absorbance values obtained at 450 nm using the standard concentration curve. Within each experiment, values from treated samples were normalized to the corresponding control samples from the same donor to reduce interindividual variability.

### 2.6. Statistical Analysis

A two-tailed Student’s *t*-test was used to evaluate the statistical significance of the differences between the means quantified for each experimental group in the series of experimental analyses described above. qRT-PCR data were analyzed by a comparative CT method; relative gene expression values (2^−ΔCt^), normalized to the reference gene *GAPDH*, were shown as mean ± SD. Statistical differences between groups were calculated with Student’s *t*-test analysis.

## 3. Results

### 3.1. MFH Significantly Reduces Positive SA-β-Gal Fibroblasts

**Figure 1:**
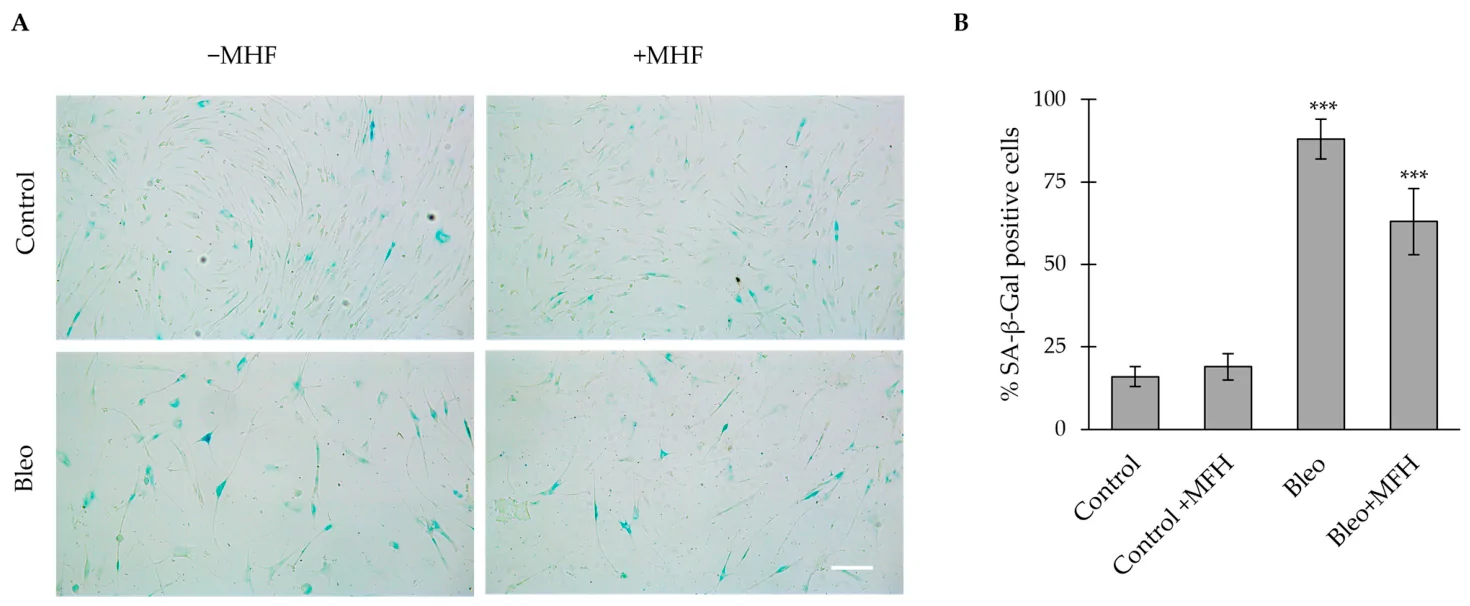
Increased SA-*β*-gal activity in primary human fibroblasts in culture treated with Bleo for 24 h, and subsequently treated in the presence or absence of MFH 0.1 mg/mL. (**A**) Representative images of bright-field microscopy of cell cultures stained to detect SA-*β*-gal activity associated with senescence. Scale: 30 µm. (**B**) Quantification of the % of cells positive for SA-*β*-gal activity under the different conditions described. The results are represented as the mean of 3 independent experiments ± SD (*** *p* < 0.001).

We first aimed to characterise the effect of MFH on premature-senescent cells. To this end, we used human primary dermal fibroblasts obtained from donors (up to 40 years). Cells were induced to enter DNA damage senescence using a well-established Bleo treatment protocol [19,20]. Treatment with Bleo 10 μM in cultured fibroblasts resulted, as expected, in a significant increase in the number of cells showing high SA-*β*-galactosidase (SA-*β*-gal) activity, a phenotype widely accepted as an indicator of entry into senescence [21], as compared to untreated control cells or control cells grown in the presence of MFH 0.1 mg/mL (Figure 1A,B).

Cells treated with Bleo for 24 h followed by MFH showed a significant number of SA-β-gal-positive cells.

### 3.2. TUNEL-Positive Fibroblasts Reflect MFH Senescent Cell Clearance Activity

**Figure 2:**
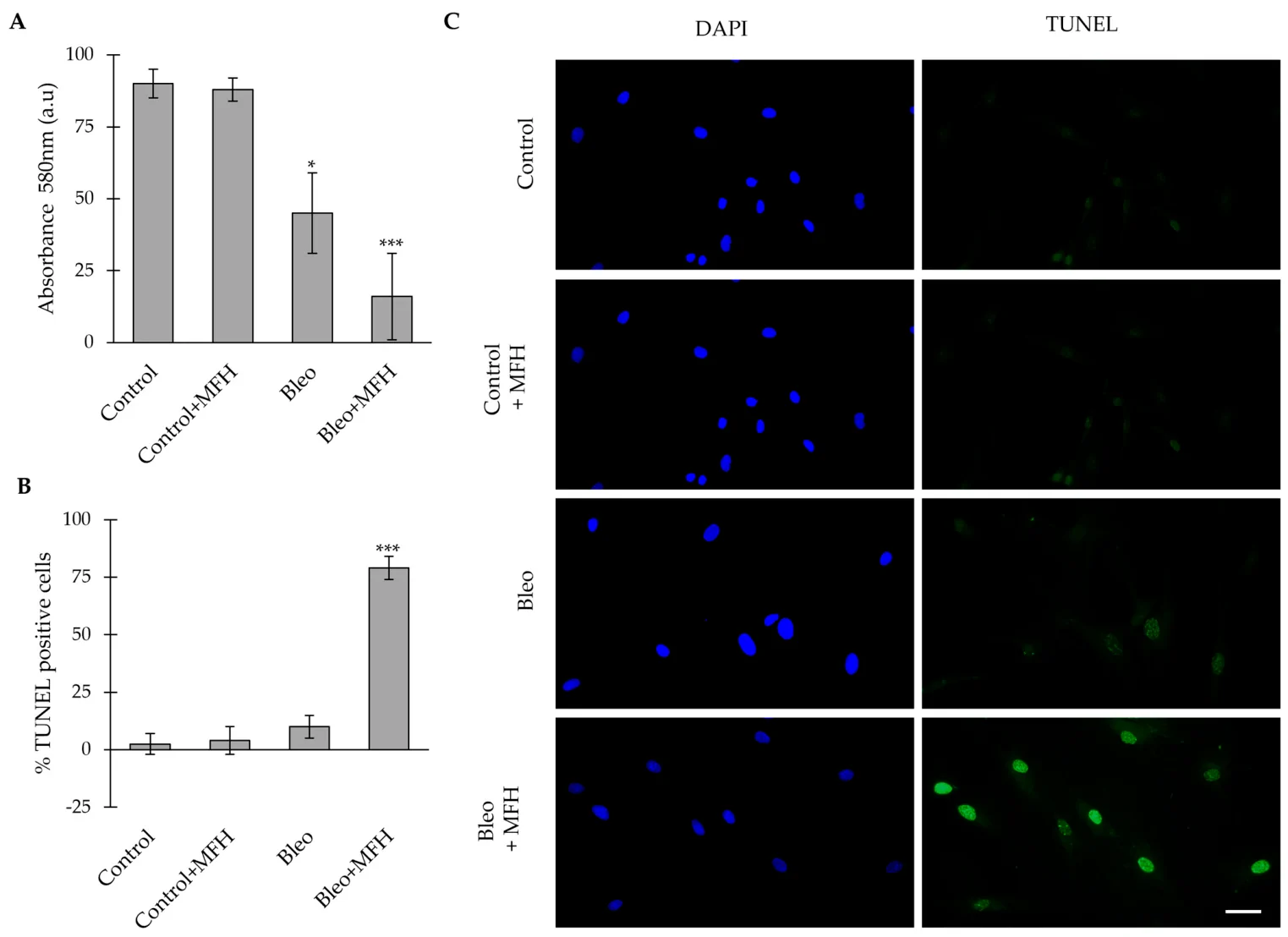
Decrease in the overall cell biomass and the activation of cell death programs in primary human fibroblasts in culture treated with 10 μM Bleo for 24 h and followed by the presence of 0.1 mg/mL MFH. Four experimental conditions were tested: control (mock treatment during 24 h), Control + MFH (mock treatment was removed after 24 h incubation and subsequently, MFH was added), Bleo (Bleo was added during 24 h of incubation and thereafter removed for fresh medium), Bleo + MFH (Bleo was added during 24 h of incubation and subsequently MFH was added). (**A**) Quantification of the number of viable cells (Crystal violet assay) after 7 days in the indicated experimental conditions. (**B**) Quantification of the number of cells identified as positive by the TUNEL technique under the indicated experimental conditions. (**C**) Representative images of fluorescence microscopy of cell cultures stained with TUNEL. Results are represented as the mean of 3 independent experiments ± SD (* *p* < 0.05; *** *p* < 0.001. Scale: 20 μm).

A significant decrease in the overall cell biomass treated with Bleo for 24 h, followed by MFH, was observed (Figure 2A), suggesting a possible effect of this compound on induced senescent cells.

We also observed that this decrease in the biomass of cells treated with Bleo for 24 h, followed by MFH, may be associated with the activation of cell death-associated DNA fragmentation, specifically as identified by the TUNEL technique (Figure 2B).

It is important to note that Bleo induces senescence through DNA damage. Therefore, TUNEL staining is not specific for apoptosis. Consequently, these preliminary findings may reflect a partial clearance of damaged cells, including senescent cells, rather than exclusively apoptotic ones.

**Figure 3:**
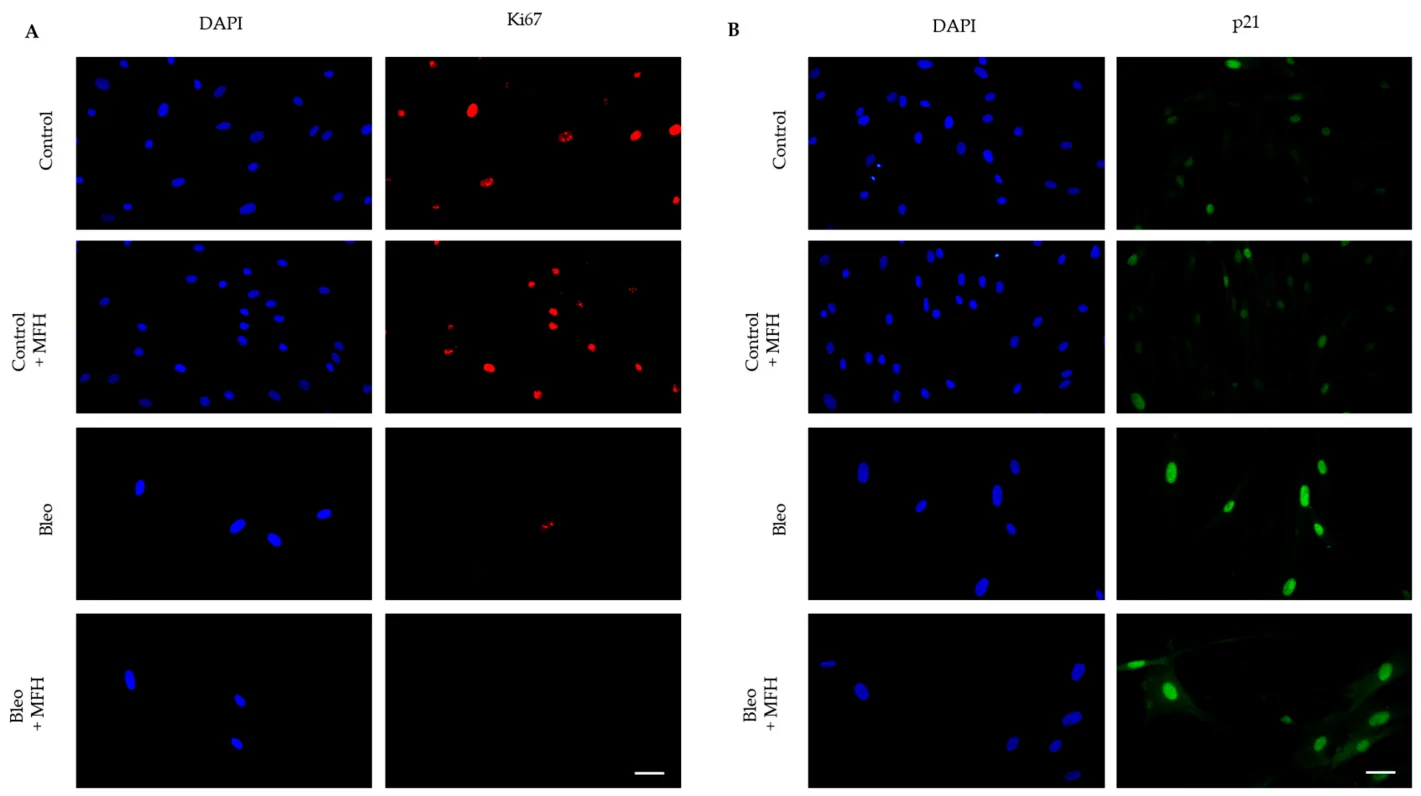
Qualitative decreased expression of the cell proliferation marker Ki-67 and increased expression of the p21 senescence marker in human primary fibroblasts in culture treated with 10 μM Bleo for 24 h, followed by +0.1 mg/mL MFH, is shown. Representative images showing the immunolocalization of the proteins Ki-67 (**A**) and p21 (**B**) in cells stained with DAPI (blue). Scale: 20 μm.

The evaluation of the Ki-67 cell proliferation molecular marker indicated that treatment of primary human fibroblasts with Bleo for 24 h, followed by the presence or absence of MFH, substantially reduces the levels of this protein (Figure 3A). In contrast, evaluation of the p21 senescence molecular marker showed that the protein levels increase after 24 h of Bleo treatment, with or without MFH (Figure 3B). The presence of p21-positive cells alongside an increase in TUNEL-positive cells after Bleo and MFH treatment suggests that senescent cell clearance is partial.

Taken together, these results suggest that MFH could partially eliminate the damage caused by bleo-induced senescence. This conclusion is supported by the marked reduction in cell biomass observed in Bleo-treated cells followed by MFH treatment, as well as by the fact that the few cells remaining under these conditions are TUNEL-positive while still expressing the senescence marker p21.

### 3.3. MFH Treatment Attenuates HFs Senescent State

Next, we aimed to evaluate the senescence-protective effect of MFH in a human mini-organ, in particular, ex vivo grown hair follicles. HFs are surrounded by fatty and dermal tissue remnants. HFs were dissected and selected using standard morphological criteria. In our experience, after growing the follicles for 24 h in 10 µM Bleo, it is essential to maintain them in fresh culture medium for 6 days to promote the expression of the SASP [22]. We saw no differences in follicle morphology despite treating the follicles with Bleo for 24 h or Bleo for 24 h + MFH (Figure S2). This result appears to be equally supported by the immunochemical analysis of E-cadherin, a protein essential in tissues that require cell–cell adhesion to maintain their structural integrity (Figure S3).

**Figure 4:**
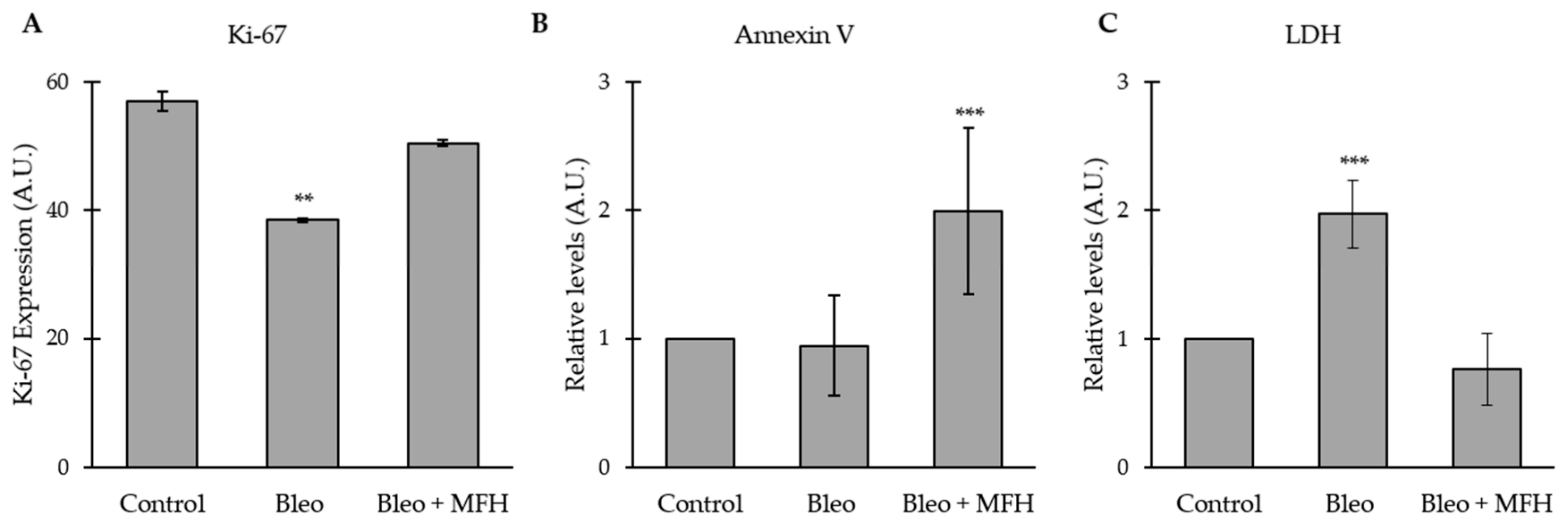
Treatment of ex vivo HFs with 10 µM Bleo for 24 h, followed by treatment with 1 mg/mL MFH, increased the proliferation marker Ki-67 levels (**A**); 1 mg/mL MFH increases the marker Annexin V levels (**B**) and 1 mg/mL MFH reduces the extracellular LDH levels (**C**). The determination of the corresponding activities was carried out by ELISA assays using extracts from a minimum of 15 HFs per condition (**A**,**B**) or supernatant of the culture medium (**C**). Statistical differences between groups were calculated with Student’s *t*-test. The bars represent the mean of all measures and the top bars ± SD (** *p* < 0.01, *** *p* < 0.001 against the control group).

On the other hand, HF treatment with Bleo resulted in lower levels of the proliferation marker Ki-67 (Figure 4A). After Bleo, the addition of MFH attenuated this overall effect. While the Bleo 24 h experimental condition did not show an increase in Annexin V protein levels, the combination of Bleo 24 h + MFH significantly increased protein levels of this marker in the tissue (Figure 4B), reinforcing the idea that MFH can act as a compound associated with senescent cell clearance in the HF. Although the observed increase in Annexin V levels may be consistent with enhanced cell death-associated processes, it should be noted that the quantification is from the lysate and does not directly assess phosphatidylserine externalization. Consequently, these findings cannot be interpreted as categorical apoptosis evidence. Nevertheless, the combination of the changes observed in Annexin V levels and the localization of DNA-damaged cells by TUNEL (Figure S4) staining in histological sections appears to be consistent with this hypothesis. Moreover, HF with Bleo induced an increase in lactate dehydrogenase (LDH) activity in the culture medium, an effect also significantly reversed by MFH (Figure 4C). It has been described that the onset of senescence promotes drastic metabolic changes, including reduced mitochondrial respiratory activity and increased anaerobic glycolysis with high lactate production associated with increased LDH activity [27,28].

**Figure 5:**
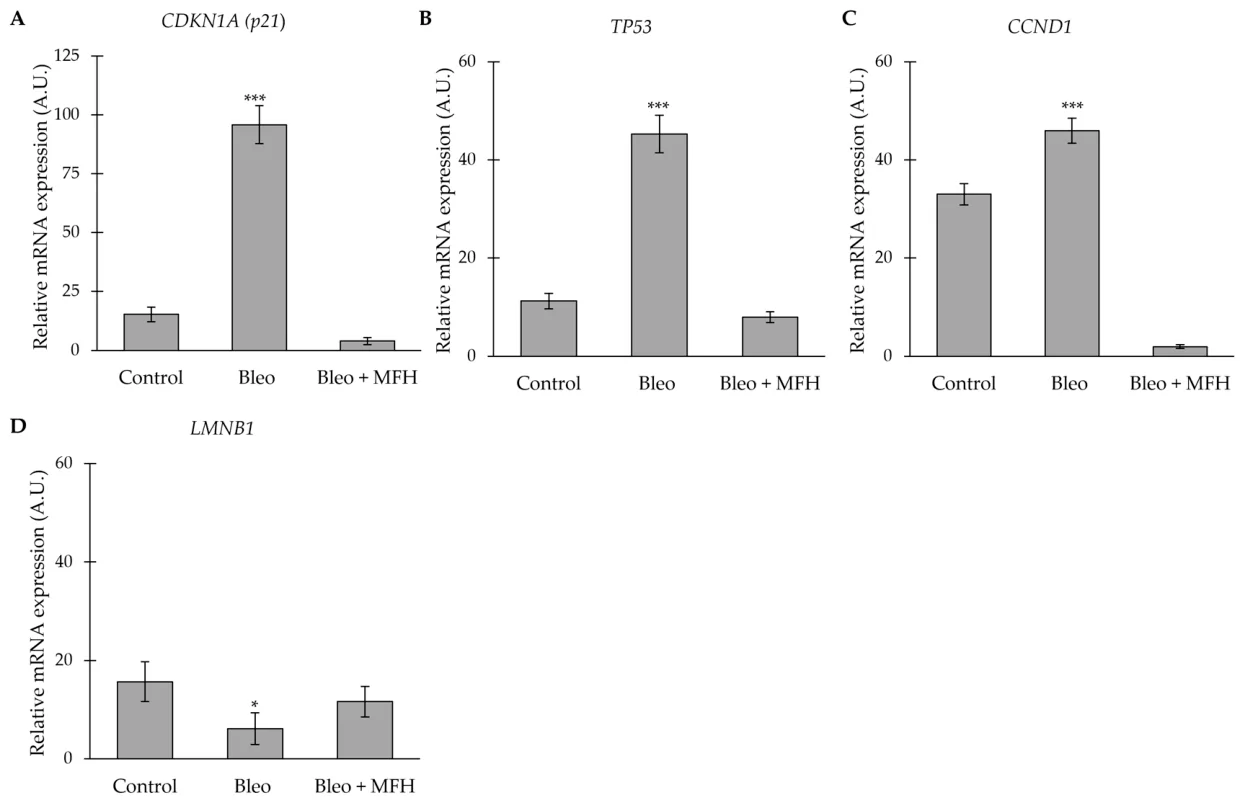
The expression of genes associated with the induction of senescence is significantly altered in ex vivo human HF treated with 10 µM Bleo for 24 h, and this effect is partially normalized in the presence of 1 mg/mL MFH. The expression of the indicated genes *CDKN1A* (p21), *TP53*, *CCND1*, and *LMNB1* was quantified by qRT-PCR from RNA extracted from a minimum of 15 HF per condition. Relative gene expression values (2^−ΔCt^), normalized to the reference gene *GAPDH*, are shown as mean ± SD. Statistical differences between groups were calculated with Student’s *t*-test analysis. (* *p* < 0.05 against the control group, *** *p* < 0.001 against the control group).

Similarly, the analysis of senescence marker genes, including *CDKN1A* (p21), *TP53*, *CCND1*, and *LMNB1* [29], showed that the treatment of HFs with Bleo for 24 h, according to the described protocol, adjusts to the senescence-associated profile in tissue, which is partially normalized in the presence of MFH (Figure 5).

The activation of cyclin D1 expression is especially significant; this protein (CCND1) is involved in the regulation of mitotic entry and accumulates in the cell during the initial phases of senescence induction, unable to activate cell proliferation due to the presence of proteins such as p16, p21, and p53 [30].

However, other marker genes involved in SASP induction, such as *CDKN2A* (p16), *IL-6,* and *ANKRD1*, did not show potential variations in Bleo-treated 24 h HF in the presence or absence of MFH compared to control samples (Figure S5). It should be noted that the transcriptional pattern conforms to the pattern observed in the senescent phenotype [31,32] and is reversed by a compound capable of making senescent cells undergo apoptosis [33], as may be the case with MFH.

Taken together, the results obtained suggest that, in the HFs grown ex vivo, MFH may attenuate the effects of Bleomycin-induced DNA damage senescence. Considering that, in cultured primary human fibroblasts, MFH appears to act as a clearance agent, the overall effect of this combination of ingredients suggests that the underlying process involves a partial removal of senescent cells within the tissue, followed by subsequent tissue restoration.

## 4. Discussion

Mammalian cells and tissues are in a continuous transition between the functional states of resilience and senescence, depending on the integration output of different damage triggers [34,35]. Resilience refers to the capacity of the tissue to return to homeostasis after internal or external damage while preserving morpho-functional identity. This critical capacity relies on a coordinated network of repairing and enabling cells not only to survive stress but to fully re-establish the homeostatic potential of the tissue. In contrast, a cellular senescence program is activated when the damage surpasses the buffering capacity of these resilience mechanisms [36].

The transition between resilience and senescence is not a binary response, but a process regulated by thresholds within all the interconnected signaling network components [34]. Redox homeostasis plays a central role. Low, tightly controlled ROS levels sustain adaptive signaling, whereas sustained oxidative stress promotes DNA damage responses and triggers senescence through p53/p21 and p16/INK4a-dependent signaling. Mitochondrial function, epigenetic plasticity, and the efficiency of proteostatic and autophagic systems further act as reinforcing molecular switches marking cells for recovery or irreversible arrest.

Cell fate determination between growth arrest and apoptosis programs largely depends on the severity of every cellular damage event experienced by the cell. Apoptosis and senescence are not fixed endpoints but dynamic states that depend on the interplay of complex signalling networks [37]. In this sense, senescence is considered a stable arrest in the G1/G2 phase of the cell cycle in which the cell is still metabolically active and unable to enter in a cell death program [29,38]. Although the underlying signaling pathways are not yet fully characterised, it is widely recognised that p53, together with p21, the BCL-2 family and imbalanced oxidative stress, which are key players in the damage response, are also major triggers of this abnormal state. In tissues, senescent cells establish a specialized microenvironment characterized not only by cell cycle arrest but also by an associated secretome SASP) and the subsequent recruitment of immune cells, which facilitates both cellular clearance and tissue regeneration [10,38,39,40].

Thus, senescent cells are characterized by the activation of specific tumor suppressor markers, including p53/p21CIP1 and p16INK4a/Rb, which inhibit cyclin-dependent kinases CDK2 (by p53/p21CIP1), CDK4 and CDK6 (by p16INK4a/Rb), resulting in the blockage of cell cycle progression [39]. Equally important, in tissues the SASP contributes to inflammation, promoting the expression of specific markers, including TNFs and ILs and macrophage recruitment [4]. The senescent phenotype also involves metabolic alterations, including an increase in glycolytic activity and L-lactate, resulting in an increased expression of LDH [40] and morphological changes in the cell [41]; however, the extracellular release of LDH may also reflect the loss of membrane integrity associated with cell death, so although it may suggest membrane alterations and damage associated with induced senescence, we cannot distinguish it from a potential cytotoxic effect. Particularly, the inner nuclear membrane, which is bound to the chromatin by the nuclear lamina, is rapidly disorganized, and the architecture of the nuclear compartment is impaired [42]. This process is associated with a Lamin B1 decrease [29]. All these markers constitute essential tools to determine and characterize the molecular entry into a senescent process and, therefore, to evaluate potential senescence-associated agents.

The results reported here suggest that MFH is a combination of natural-origin ingredients that partially eliminate cells that have entered DNA-damage senescence. This observation is suggested by the fact that a significant decrease in the overall biomass of cells in cultures treated with Bleo 24 h occurs specifically in the presence of MFH, and that the few cells that remain and grow under these conditions are positive for the TUNEL marker and continue to express the senescence marker p21CIP1. Thus, considering the results obtained in primary fibroblasts, we also decided to evaluate the effects of MFH on human hair follicles obtained from donors induced to enter DNA-damage senescence using Bleo treatments. HFs can be considered a complete mini-organ that, due to their well-defined physiology and relative ease of manipulation, is widely used as an ex vivo experimental model to test new drugs and dermocosmetic compounds [43].

Our results showed that MFH may attenuate the effects of chemically induced senescence entry in HFs grown ex vivo. Bleo treatment of cultured HFs resulted in a significant reduction in the cell proliferation marker Ki-67 and a strong induction of the expression of key gene markers of the senescent phenotype, including *p53*, *p21CIP1*, and *CCND1*, together with inhibition of *LMNB1* expression, indicating the entrance of the tissue into a senescent state. In addition, an increase in LDH activity was also observed in Bleo-treated HFs, indicating a possible metabolic alteration in the tissue or, in fact, a cytotoxic reaction due to Bleo. These effects were effectively attenuated in the presence of MFH. It can be hypothesized that the increased LDH levels induced upon entry into senescence may result in their extracellular release via vesicular pathways [44]. Although LDH activity is not regarded as a canonical marker of senescence, it has been associated with the senescent state [45]. Notably, Bleo-induced senescent HFs treated with MFH showed an increase in apoptotic markers (Annexin V and TUNEL), but also an overall reactivation of Ki-67 expression in the HF. Considering that, in cultured primary human fibroblasts, MFH appears to act as a potential senescence-associated agent, the observation that the overall effect of this molecule in a mini-organ such as the HF is to attenuate the senescent phenotype. We hypothesise that this process involves a partial clearance within the tissue, potentially followed by a regenerative response driven by non-senescent cell populations, which may contribute to the maintenance of tissue homeostasis.

Further progression of this work will require additional replicates to strengthen the statistical support for these findings. Additionally, the evaluation of inflammatory markers would be valuable to define the SASP framework within this specific ex vivo model, HFs. It is important to note that the collection and processing of HFs constitute a limitation of this study, restricting both the number of biological replicates and the range of experimental conditions that could be evaluated. Future studies should incorporate additional control groups to strengthen the robustness of the findings. Furthermore, the Bleo-based model of DNA damage-induced senescence highlights an important limitation in interpretation of the results. In this context, markers such as Annexin V and LDH release may potentially confound the analysis by supporting the hypothesis of a partial elimination of senescent cells, while actually reflecting the cytotoxic effects of the senescence-inducing agent. Consequently, the possibility remains that the observed effects reflect a combination of senescent cell clearance and nonspecific cytotoxicity induced by the senescence trigger. Another limitation of the present study is that formulation optimization, effective dermal and follicular delivery, and in vivo validation were beyond the scope of this work. These represent important next steps toward evaluating its therapeutic applicability. Likewise, the efficacy of the individual ingredients was not an objective of this study. Nevertheless, future investigation of each ingredient separately, together with their analytical characterization, could provide valuable information.

Finally, to reinforce the findings presented here, it will be essential to benchmark the senescent cell clearance activity of the investigated ingredient against compounds already established as senolytics, thereby enabling a clearer contextualization of its relative efficacy.

In this context, senotherapeutic agents have emerged as potential modulators of the resilience/senescence transition [34,35,46]. Several naturally derived compounds with established redox-modulating properties exhibit dual roles at the interface of resilience and senescence. These molecules can reinforce cellular resilience by promoting adaptive stress responses and maintaining redox balance [47,48]. However, under specific stressful conditions or in senescent cells, they may promote a shift toward senolytic or senomorphic activity [48]. At the tissue level, the interplay between resilience-enhancing mechanisms and a senotherapeutic treatment may also critically modulate the differential activation of aging or regeneration processes, paving the way for future therapeutic approaches to reinforce tissue homeostasis and delay degenerative processes. In this regard, although, as has already been described throughout this paper, we do not have sufficient evidence to classify MFH as a senotherapeutic agent for HFs, these results are sufficient to warrant further research aimed at classifying the ingredient as a potential naturally occurring senolytic.

## Figures and Tables

**Table 1:** Primer sequences used for gene expression quantification by qRT-PCR.

Gene	Forward 5′–3′	Reverse 5′–3′
*ANKRD1* [23]	AGT AGA GGA ACT GGT CACTGG	TGG GCT AGA AGT GTCTTC AGA T
*CDKN1A* (p21) [23]	GAC ACCACT GGA GGG TGA CT	CAGGTC CAC ATG GTC TTC CT
*CDKN2A* (p16) [23]	CCAACGCACCGAATAGTTACG	GCGCTGCCCATCATCATG
*IL-6* [23]	CCGGGAACGAAAGAGAAGCT	GCGCTTGTGGAGAAGGAGTT
*TP53* [24]	AAG AAACCA CTG GAT GGA GAA	CAG CTC TCG GAA CAT CTC GAA
*CCND1* [25]	TGAAGGAGACCATCCCCCTG	TGTTCAATGAAATCGTGCGG
*LMNB1* [26]	GTATGAAGAGGAGATTAACGAGAC	TACTCAATTTGACGCCCAG
*GAPDH* [26]	AGCCACATCGCTCAGACAC	GCCCAATACGACCAAATCC

## Data Availability

The raw data supporting the conclusions of this article will be made available by the authors on request. The specific combination of *Galega officinalis* extract, *Hippophae rhamnoides* fruit oil, and resveratrol is under patent (WO 2025/181399) and licensed by Cantabria Labs.

## Supplementary Materials

The following supporting information can be downloaded at https://www.mdpi.com/article/10.3390/cimb48101041/s1.

## Abbreviations

The following abbreviations are used in this manuscript:

*β*-Gal	*β*-Galactosidase
Bleo	Bleomycin
HFs	Hair follicles
LDH	Lactate dehydrogenase
MFH	Metforhair^®^
OS	Oxidative stress
SASP	Senescence-associated secretory phenotype
