# Supplementary material for: A Novel Combination of Galega officinalis Extract, Hippophae rhamnoides Fruit Oil and Resveratrol Attenuates Bleomycin-Induced Senescence Markers in Human Dermal Fibroblasts and Ex Vivo Hair Follicles
Source: Curr Issues Mol Biol. 2026 Oct 8;48(10):1041. doi: 10.3390/cimb48101041 (PMC13703739; doi:10.3390/cimb48101041)
Supplement: Supplementary file 1 [file cimb-48-01041-s001.zip › cimb-4546827-supplementary.pdf]

current issues in
molecular biology

MDPI

Supplementary Materials

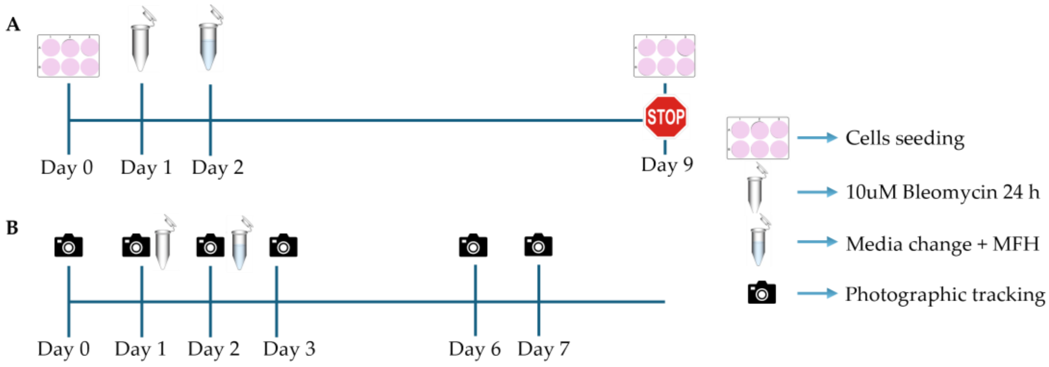

Figure S1. Schematic representation of the protocol for senescence induction with 10 µM Bleo in fibroblasts (A) and in *ex vivo* HFs (B). Schematic created using PowerPoint.

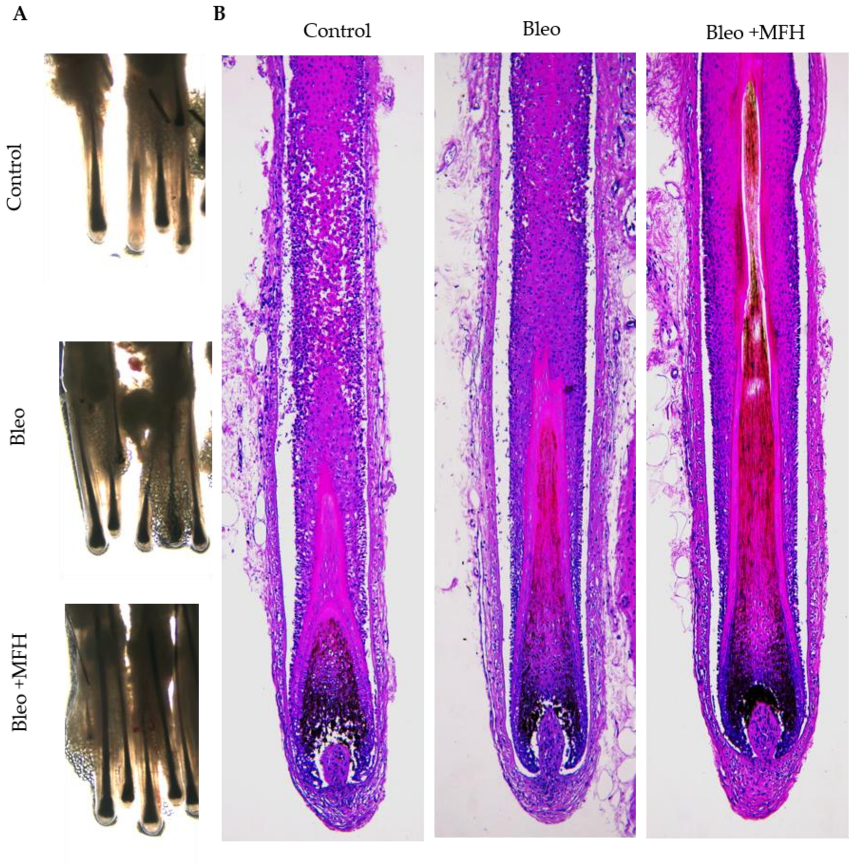

Figure S2. Treatment of *ex vivo* HFs with 10 µM Bleo 24 h, followed by the absence or presence of 1 mg/mL MFH does not significantly alter their histomorphological structure. Representative images are shown in bright-field microscopy (**A**) and in histological sections stained with H&E (**B**). Scale bar: 100µm.

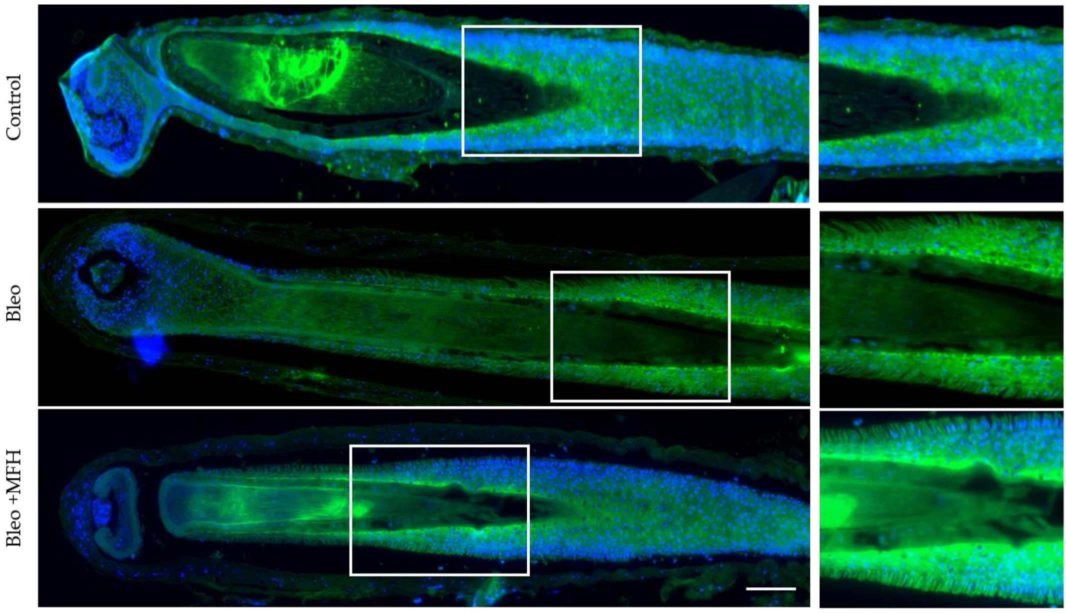

Figure S3. Treatment of *ex vivo* HF with 10 µM Bleo for 24 h, followed by the presence or absence of MFH does not appear to alter the expression and/or distribution of E-cadherin, tissue structural marker. Representative images of protein immunolocalization in histological sections of the tissue under the indicated treatment conditions. The right panel shows magnifications of the regions boxed in the left panels. Scale bar: 100µm.

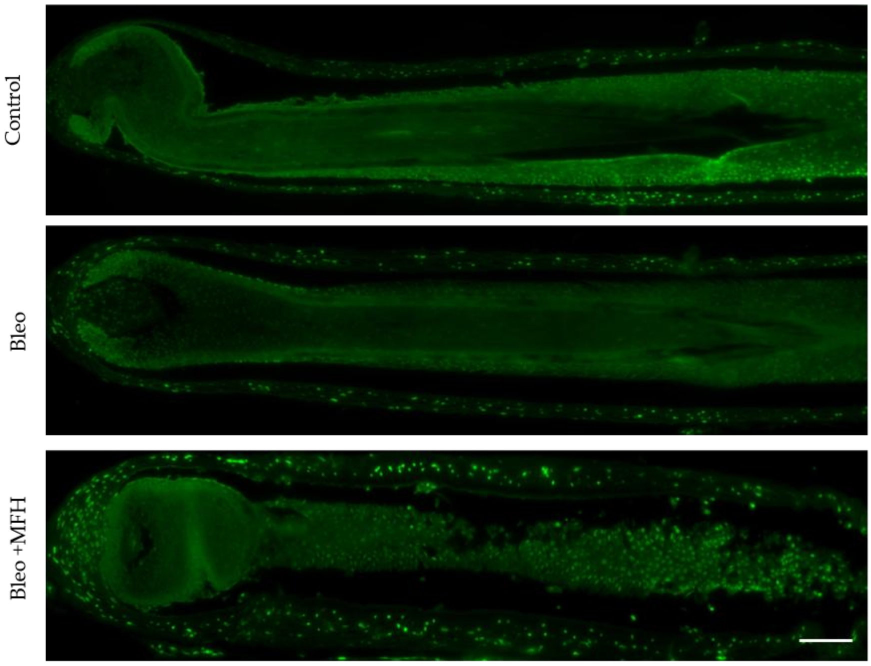

Figure S4. Treatment of *ex vivo* HF with 10 µM Bleo for 24 h, followed by the presence or absence of MFH appears to increase the number of TUNEL-positive cells in the tissue. Representative images of protein immunolocalization in histological sections of the tissue under the indicated treatment conditions. Scale bar: 100µm.

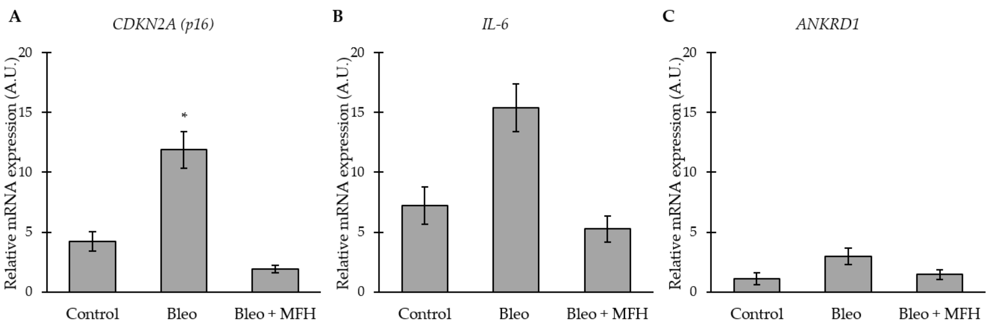

Figure S5. The expression of the senescence-inducing genes *CDKN2A* (*p16), IL-6*, and *ANKRD1*, associated with the SASP. The expression of these genes was quantified by qRT-PCR using RNA extracted from a minimum of 15 HF per condition. With 10 μM Bleo for 24 h, followed by the presence of MFH, (A) *CDKN2A (p16),* (B) *IL-6,* and (C) *ANKRD1* displayed expression levels approaching those of the control group.Relative gene expression values ($2^{-\Delta Ct}$), normalized to the reference gene GADPH, are shown as mean ± SD. Statistical differences between groups were calculated with Student’s t-test analysis. (* $p < 0.05$ against the control group).
